# Genome-Scale Metabolic Network Reconstruction and In Silico Analysis of Hexanoic acid Producing *Megasphaera elsdenii*

**DOI:** 10.3390/microorganisms8040539

**Published:** 2020-04-09

**Authors:** Na-Rae Lee, Choong Hwan Lee, Dong-Yup Lee, Jin-Byung Park

**Affiliations:** 1 Department of Bioscience and Biotechnology, Konkuk University, Seoul 05029, Korea; michelle3690@gmail.com (N.-R.L.); chlee123@konkuk.ac.kr (C.H.L.); 2 Department of Food Science and Engineering, Ewha Womans University, Seoul 03760, Korea; 3 School of Chemical Engineering, Sungkyunkwan University, 2066 Seobu-ro, Jangan-gu, Suwon, Gyeonggi-do 16419, Korea

**Keywords:** *Megasphaera elsdenii*, hexanoic acid, bifurcated pathway, genome-scale metabolic model, constraint-based modeling

## Abstract

Hexanoic acid and its derivatives have been recently recognized as value-added materials and can be synthesized by several microbes. Of them, *Megasphaera elsdenii* has been considered as an interesting hexanoic acid producer because of its capability to utilize a variety of carbons sources. However, the cellular metabolism and physiology of *M. elsdenii* still remain uncharacterized. Therefore, in order to better understand hexanoic acid synthetic metabolism in *M. elsdenii*, we newly reconstructed its genome-scale metabolic model, *i*ME375, which accounts for 375 genes, 521 reactions, and 443 metabolites. A constraint-based analysis was then employed to evaluate cell growth under various conditions. Subsequently, a flux ratio analysis was conducted to understand the mechanism of bifurcated hexanoic acid synthetic pathways, including the typical fatty acid synthetic pathway via acetyl-CoA and the TCA cycle in a counterclockwise direction through succinate. The resultant metabolic states showed that the highest hexanoic acid production could be achieved when the balanced fractional contribution via acetyl-CoA and succinate in reductive TCA cycle was formed in various cell growth rates. The highest hexanoic acid production was maintained in the most perturbed flux ratio, as phosphoenolpyruvate carboxykinase (*pck*) enables the bifurcated pathway to form consistent fluxes. Finally, organic acid consuming simulations suggested that succinate can increase both biomass formation and hexanoic acid production.

## 1. Introduction

Hexanoic acid and its derivatives are generally used for the production of artificial flavors and food additives, as well as plastics, rubber, transportation fuel, and pharmaceuticals [1,2,3,4]. Although they are mainly produced via the multi-step chemical conversion of petroleum products and natural oils, recently, microbial cell factories have been considered as a promising approach for the sustained production of this straight six-carbon chain carboxylic acid. For example, *Clostridium kluyveri* synthesizes hexanoic acid by fermenting ethanol with acetate or succinate [5]. *Rhodospirillum rubrum* and *Clostridium scatologenes* are other hexanoic acid producers utilizing pyruvic acid and methanol as carbon sources, respectively [6,7]. Furthermore, hexanoic acid can be also produced in *Megasphaera elsdenii* fueled by glucose, maltose, and sucrose. Among such potential producers, *M. elsdenii* has noticeable advantages, as it can consume diverse carbohydrates such as starch, mannitol, glucose, maltose, and sucrose, which are economic carbon sources [8], and also achieve higher titre of hexanoic acid compared with other candidates, namely, 19 g/L and 28.42 g/L from glucose [9] and sucrose [1], respectively.

In order to enhance the titre further toward an industrial scale, it is highly required to systematically understand the cellular behavior under various environmental and genetic conditions, as well as the relevant metabolic states to the hexanoic acid synthetic pathway in *M. elsdenii*, which is anaerobic Gram-negative bacterium found in rumen of cattle, sheep, and the human gut. In this regard, constraint-based metabolic modeling and *in silico* analysis have been appreciated as one of the attractive techniques to characterize it; a multitude of genome-scale metabolic models (GSMMs) have been built for elucidating the cellular physiology of various organisms. However, only a handful of GSMMs for volatile fatty acid (VFA) derivative producers are available to date, including *Clostridium acetobutylicum,* producing butyric acid and butanol [10], and *Clostridium beijerinckii* for butanol production [3]. Both models have been used to explain the biosynthetic pathways of C4 metabolites (butyric acid and butanol) in *Clostridium spp*. Thus, we believe anaerobic bacteria producing higher value C6 compounds (hexanoic acid and hexanol) can be further characterized by resorting to this *in silico* modeling approach. To this end, we reconstructed a manually curated GSMM of *M. elsdenii* based on its genome annotation, which is poorly studied, although it has high biotechnological potentials [11]. Subsequently, a flux balance analysis (FBA) was applied to investigate the mechanism for C6 compounds production and relevant pathways in the microorganism.

## 2. Materials and Methods 

### 2.1. Genome-Scale Metabolic Model Reconstruction 

The genome-scale metabolic network model of *M. elsdenii* was reconstructed based on a genome sequence of *M. elsdenii* DSM20460 [11] by using various genomic and biological databases following the established procedure [12]. An initial network was drafted by compiling annotated metabolic genes and relevant biochemical reactions from KEGG [13] and Biocyc [14]. Then, the constructed draft model was further refined through manual curation by correcting elemental imbalances, matching genes to appropriate biochemical reactions, and filling the gaps. In addition, non-gene-associated reactions such as metabolite transport were included in the model according to databases and literatures [8,13,14]. The network connectivity was also examined by maximizing the production of each metabolite component in the biomass equation or by-products such as acrylic acid, butyric acid, and hexanoic acid from various carbon sources, including lactic acid and glucose. The identified missing links were added with necessary reactions to fill the gaps.

### 2.2. Biomass Composition

The formulation of the biomass, which is an important prerequisite for *in silico* flux analysis, is composed of several building blocks such as amino acids, nucleic acids, and fatty acids. The building block synthetic reactions represent polymerizations of each metabolite precursor. The amino acids and phospholipid compositions were obtained from previous studies [15,16], while the compositions of DNA and RNA were calculated based on their reported G+C content of 52.8% [11]. The overall biomass composition was adopted from *E. coli* [17]. Detailed information about calculating the biomass composition is provided in Supplementary Data S1. 

### 2.3. Constraint-Based Flux Analysis

We simulated the cellular metabolism of *M. elsdenii* by employing constraint-based flux analysis under various culture conditions, as described elsewhere [18,19]. The biomass reaction was maximized to evaluate the cell growth during the exponential phase. In order to solve the optimization mathematically, the objective function, namely the biomass formation rate, was set to be maximized, and was subjected to stoichiometric and capacity constraints in a linear programming formulation, as follows:

$$\max Z=\sum_{j}^{}c_{j}v_{j}$$

Subject to the following:

$$\sum_{j}^{}S_{ij}v_{j}=0$$

$$v_{j}^{\min}\leq v_{j}\leq v_{j}^{\max}$$

where *S_ij_* is the stoichiometric coefficient of metabolite *i* involved in reaction *j*; *v_j_* refers to the specific rate of metabolic reaction *j*; and the reaction capacity is constrained using parameters *v_j_^min^* and *v_j_^max^*, representing the lower and upper bounds of reaction *j*, respectively. We describe *v_j_^min^* = −inf and *v_j_^max^* = inf as reversible reactions, or *v_j_^min^* = 0 and *v_j_^max^* = inf as irreversible reactions. *Z* denotes the cellular objective of all of the metabolic reactions, where the relative fluxes are determined by coefficient *c_j_*. The COBRA toolbox was used to solve the constraint-based flux analysis [20].

### 2.4. Gene Essentiality Analysis

The gene essentiality analysis was conducted by evaluating the maximal biomass formation rate while constraining the relevant reactions to be zero under a defined glucose uptake rate. The gene is classified to be essential for cell growth when the *in silico* resulting cell growth of the mutant is zero. The gene essentiality test was carried out using the COBRA toolbox [20].

### 2.5. Flux Ratio Analysis

In order to examine the fractional contribution for hexanoic acid production in a furcated pathway, we performed a flux ratio analysis using the COBRA toolbox. First, the model was simulated to investigate the flux distribution of the bifurcated pathway with hexanoic acid production in different cell growing states, by fixing the specific glucose uptake rate and growth rate while setting the hexanoic acid synthetic reaction as the objective function. Subsequently, in each specific growth rate, nine different flux ratios (Route A:Route B = 10:1, 5:1, 3:1, 2:1, 1:1, 1:2, 1:3, 1:5, and 1:10) were tested to check for changes in the flux distribution. 

## 3. Results and Discussion

### 3.1. Reconstruction of M. elsdenii Metabolic Network Model

The genome-scale metabolic network of *M. elsdenii* (*i*ME375) was reconstructed, followed by model refinement. A number of network gaps were filled by adding suitable metabolic reactions based on the information obtained from metabolic pathway databases (e.g., Biocyc) and literature sources. For example, the draft model contained a few gaps in the synthetic pathway of propionic acid, which is the major fermentation product from lactate in *M. elsdenii.* Thus, we added the reactions corresponding to lactate racemase and lactoyl-CoA dehydratase for the conversion of d-lactate into l-lactate, and the dehydration of lactoyl-CoA to acryloyl-CoA, respectively, according to previous studies [21,22]. The gap filling of the draft model was also conducted via a sequence-based homology test utilizing BLASTp in the NCBI database by identifying evidence for the newly added enzymes. We assigned 11 putative enzymes, suggesting their new annotation (Supplementary Data S2). The initial model also had several gaps in the metabolite transportation pathways, indicating no uptake of carbon sources such as maltose, glucose, lactate, and acrylate. As previous studies have reported that *M. elsdenii* is able to grow on all of these carbon sources, we added new transport reactions based on the information available from the KEGG and Biocyc databases. 

**Figure 1:**
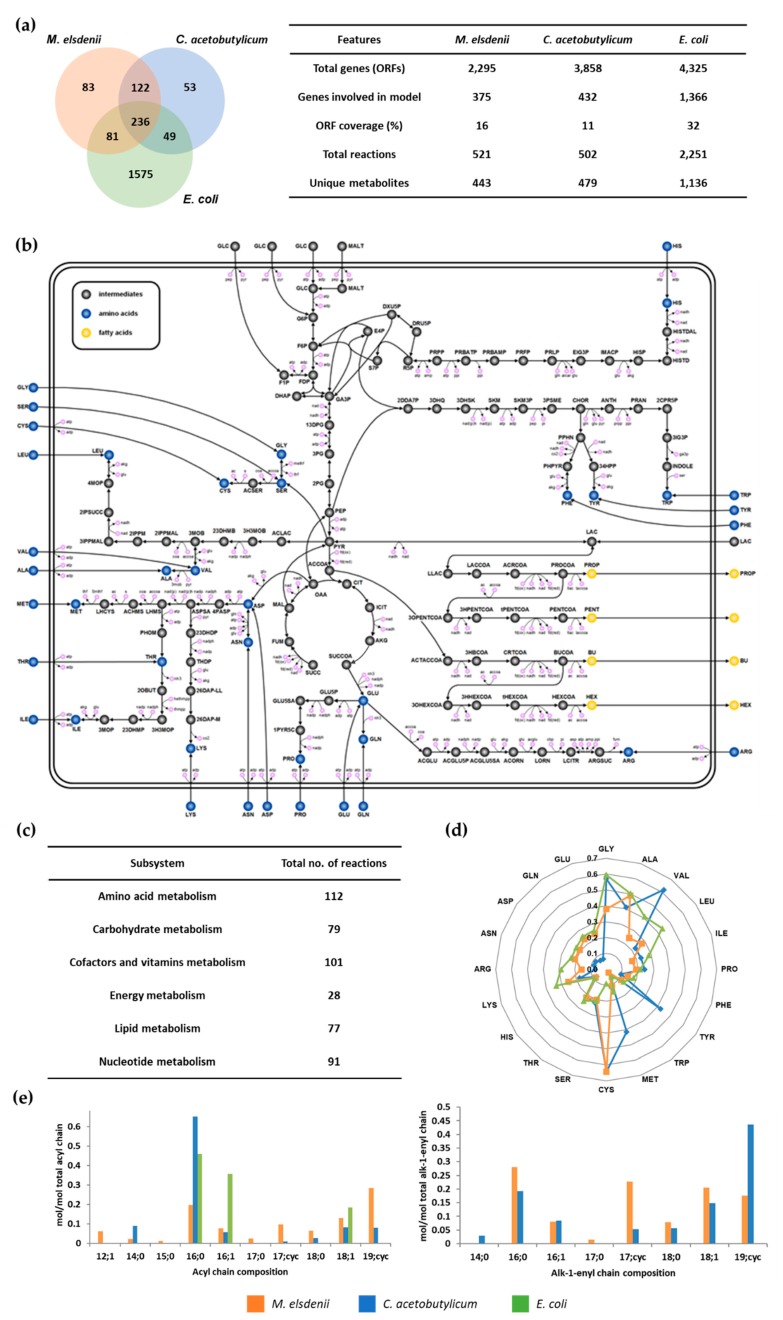
Comparison of metabolic network models and biomass compositions. (**a**) The metabolic reactions of *i*ME375 are compared with *C. acetobutylicum Cac*MBEL489 and *E. coli i*JO1366. The numbers in the Venn diagram represent the number of metabolic reactions that are common and unique to the respective organisms. (**b**) The central metabolic network of *M. elsdenii*. (**c**) Distribution of reactions across various metabolic subsystems in *i*ME375. (**d**) Amino acid composition (mmol/g protein) of three different *in silico* models. (**e**) Fatty acid composition (mmol/mol total acyl or alk-1-enyl chain fatty acids) of three different *in silico* models.

Figure 1 illustrates the overall metabolic pathways of *M. elsdenii*, including the glycolysis, tricarboxylic acid (TCA) cycle, pentose phosphate pathway (PPP), amino acids, and fatty acids synthetic pathways. Notably, the glycolysis and PPP are complete, but the TCA cycle is incomplete, as the gene of 2-oxoglutarate dehydrogenase was not found. The synthetic pathways of 20 amino acids and VFAs are also depicted in Figure 1b.

The resulting metabolic model of *M. elsdenii*, *i*ME375, accounts for 375 genes (ORF coverage; 16%), 521 metabolic reactions, and 443 metabolites. In *i*ME375, all of the 521 reactions were classified into six major subsystems, namely: amino acids, carbohydrates, cofactors and vitamins, energy, lipids, and nucleotides metabolisms. Among them, amino acids metabolism constitutes the largest part of metabolic reactions, followed by cofactors and vitamins metabolisms (Figure 1c). Detailed information containing the reactions, enzymes, genes, and metabolites of *i*ME375 is available in Supplementary Data S3. The model is also available as a Systems Biology Markup Language (SBML) file (level2, version 1, http://sbml.org/; in Supplementary Data S4).

### 3.2. Network Characteristics of iME375 and Its Comparison with C. acetobutylicum and E. coli

In order to understand the metabolic network of *M. elsdenii*, *i*ME375 was compared with the models for *C. acetobutylicum* and *E. coli,* which are *Cac*MBEL489 and *i*JO1366, respectively (Figure 1a). We used them for this comparative study, as *Cac*MBEL489 is one of the well-known metabolic models for the VFA synthesizing prokaryote, and *i*JO1366 is a well-developed prokaryote model describing common bacteria physiologies. From the comparison, *i*ME375 has 236 metabolic reactions in common with *Cac*MBEL489 and *i*JO1366. Expectedly, the most common reactions belong to a central metabolism, such as glycolysis, as well as the TCA cycle and amino acids synthetic pathway. However, conspicuous differences were observed in VFAs and their derivatives synthetic pathways (Figure 1b). For example, propionic acid is synthesized from lactate via propionyl-CoA transferase in *M. elsdenii,* while *C. acetobutylicum* produces propionic acid from threonine. Furthermore, acetone and butanol are produced in *C. acetobutylicum*, whereas *M. elsdenii* is unable to synthesize them as it does not contain relevant enzymes.

The VFA synthetic pathways of *M. elsdenii* were compared with *C. acetobutylicum* in order to understand the butyric and hexanoic acid synthetic metabolism in detail (Supplementary Data S5). The homology test of the butyric and hexanoic acid synthetic enzymes in both microorganisms was conducted by a BLASTp search in the NCBI database. Interestingly, *C. acetobutylicum* does not possess acetyl-CoA hydrolase/transferase (*cat*), NADH:ferredoxin oxidoreductase (*rnf*A-E) and 4-hydroxybutyryl-CoA dehydratase (*abf*D) as compared to *M. elsdenii*. On the other hand, *C. acetobutylicum* has butyrate–acetoacetate CoA transferase (CA_P0163 and 0164) instead of acetyl-CoA hydrolase/transferase. As such, the coenzyme A (CoA) of butyryl-CoA is transferred to acetoacetate and the carbon chain elongation is terminated. In *M. elsdenii*, the butyryl-CoA (C4) is elongated to 3-oxohexanoyl-CoA (C6) by transferring the acetyl (C2) group from acetyl-CoA via acetyl-CoA C-acetyltransferase, or is converted to butyric acid by utilizing acetate (C2) as the CoA acceptor via acetyl-CoA hydrolase/transferase. However, *C. acetobutylicum* produces butyric acid (C4) and butanol (C4) through butyrate-acetoacetate CoA transferase and butanol dehydrogenase during acidogenic and solventogenic phases, respectively. Unlike *M. elsdenii*, acetoacetate (C4) is involved in CoA transferase in *C. acetobutylicum*. Thus, we suggest that the existence of the three enzymes (*cat*, *rnf*A-E, and *abf*D) and the substrate specificity of other enzymes, such as *thl*A, *hbd*, *crt,* and *cat,* which contribute to acyl-CoA elongation, may affect the carbon length of the final product in *M. elsdenii*. 

Interestingly, there are notable characteristics in the cellular composition. For example, the composition of amino acids in *M. elsdenii* is closer to *E. coli* than *C. acetobutylicum* (Figure 1d). In particular, the composition of methionine, tyrosine, valine, glutamate, glutamine, aspartate, and asparagine in *M. elsdenii* is very different from *C. acetobutylicum*. Intriguingly, the phospholipids in *M. elsdenii* and *C. acetobutylicum* consist of plasmalogen, which is usually found in human tissue. However, the types of fatty acid, which is part of plasmalogen, are different between *M. elsdenii* and *C. acetobutylicum*. *M. elsdenii* contains fatty acids with an odd number of carbons, such as 15:0 and 17:0, and medium chain unsaturated fatty acid, 12:1, which are not present in *C. acetobutylicum*. In *C. acetobutylicum,* 16:0 in acyl chain and 16:cyc in alk-1-enyl fatty acid are major components, while *M. elsdenii* contains almost a similar amount of each fatty acid in the phospholipid (Figure 1e). We included the biomass equation in *i*ME375 by considering the above-mentioned cellular physiologies of *M. elsdenii*. The biomass composition of *i*ME375 is fully explained in Supplementary Data S1. 

### 3.3. Model Validation

In order to validate the *i*ME375 metabolic network model, we examined the differences between the simulated cellular phenotypes and the experimentally observed data, which were obtained from three sets of fermentation under different media conditions—glucose containing complex medium [23], lactate containing minimal medium [24], and complex medium with lactate [22]. The biomass formation flux was maximized while constraining the carbon uptake rates that were observed in the exponential phase, as it has been hypothesized that the wild-type organisms evolve toward the maximum cell growth during the growing phase [25].

The exchange fluxes of essential minerals and nutrients such as NO_2_, NH_3_, H_2_O, SO_4_, and phosphate were unconstrained for cell growth. Additionally, VFA exchange fluxes were constrained as they measured the secretion rate in each simulation. To mimic the glucose medium condition, the reaction of lactate racemase, which converts D-lactate into L-lactate, was blocked, as the enzyme expression is induced only under lactate-containing conditions [26]. The growth associated maintenance (GAM) value, 40 mmol g^−1^ DCW h^−1^, was adopted from the GSMM of *C. acetobutylicum,* which is another VFA synthesizing microbe. In addition, we used 3.5 mmol g^−1^ DCW h^−1^ for NGAM (non-growth associated maintenance) in *M. elsdenii,* according to a previous study [15]. 

To minimize the influence of other nutrients in the culture medium, such as amino acids or trace amounts of carbon sources, which can help cell growth during the early exponential phase, we used experimental data during the late exponential phase in three different experiments for simulation. Remarkably, all of the simulated biomass formation rates were highly consistent with the observed growth rates, within the acceptable error range of 10% (Table 1).

### 3.4. Gene Essntiality Analysis

We conducted a gene essentiality analysis of *M. elsdenii* under a glucose minimal medium condition by deleting each gene at a time (see Methods), allowing us to identify lethal genes for further genetic engineering in order to enhance productivity. The genes were classified into the following three types: completely essential genes (genes that are required for cellular growth), partially essential genes (genes that are required for cellular growth, but if they are deleted they show a lower cell growth rate), and non-essential genes (genes that are not required for cellular growth). There were 221 and 24 reactions, corresponding to 182 and 21 genes, that were predicted to be essential and partially essential, respectively. Most of the amino acid, riboflavin, and coenzyme A synthetic genes are completely essential. Interestingly, VFA synthetic genes are non-essential or partially essential, as most VFA forming reactions are catalyzed by isozymes. Hexanoic acid synthetic reactions via succinate, acetate formation reactions, and butyryl-CoA:acetate CoA transferase are partially essential. In anaerobic microorganisms, acetate is known as one of the key metabolites that can accept electrons from other redox cofactors involved in butyric and hexanoic acid synthesis [27]. Like other anaerobes, *M. elsdenii* also requires acetate and protons as electron acceptors to generate energy and balance the intracellular redox potential by converting them to acetyl-CoA and H_2_, respectively [28].

### 3.5. Characteristics of Volatile Fatty Acid Synthetic Metabolism

**Figure 2:**
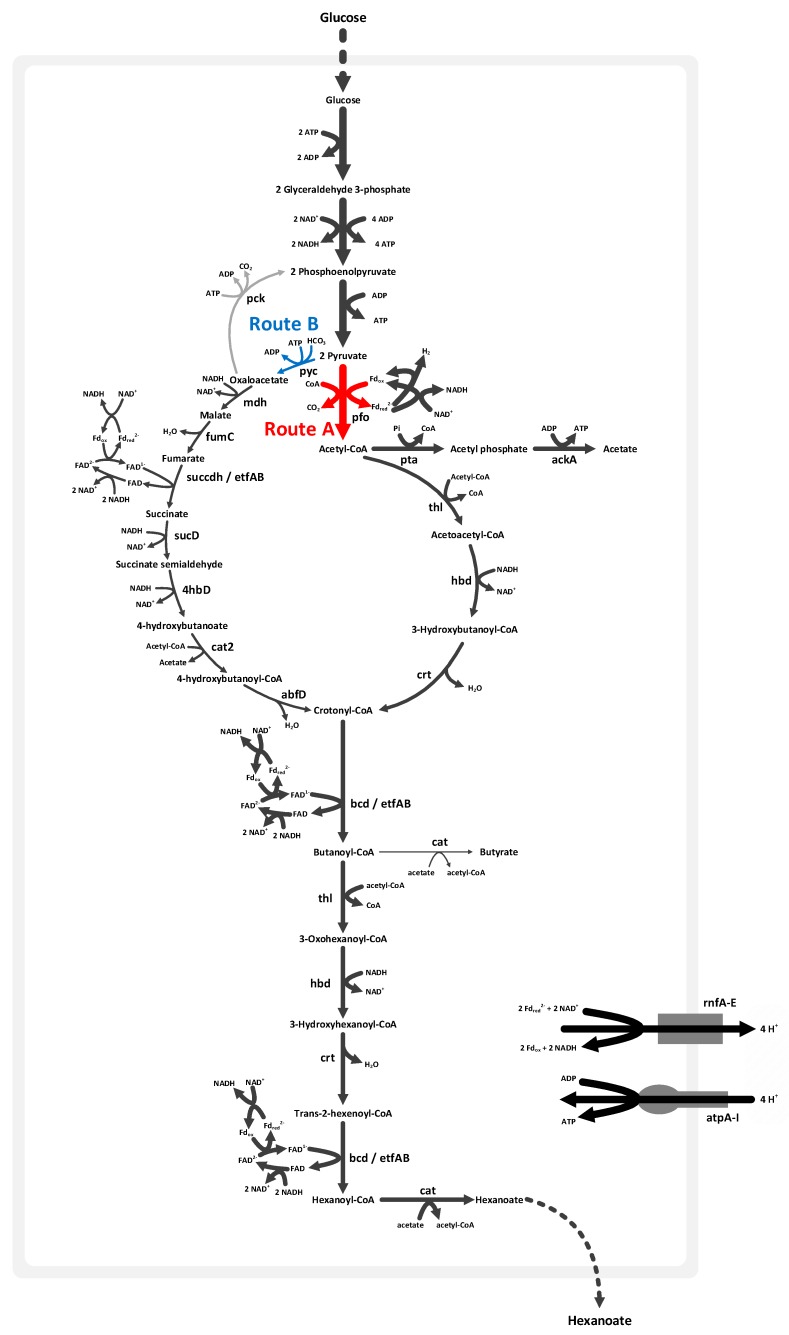
Bifurcated pathway to synthesize hexanoic acid in *M. elsdenii*. A fatty acid synthetic pathway starts from Route A through acetyl-CoA, or Route B via oxaloacetate, by running the tricarboxylic acid (TCA) cycle in a reductive direction. The thickness of the arrows represents flux values when maximizing hexanoic acid production from glucose.

Figure 2 illustrates the butyric and hexanoic acid synthetic pathways in *M. elsdenii*. We found two possible routes via acetyl-CoA (Route A) and oxaloacetate (Route B), in order to convert from pyruvate to crotonyl-CoA, which is further catalyzed to synthesize hexanoic acid. Route A is initiated with the oxidative decarboxylation of pyruvate to acetyl-CoA and CO_2_ by utilizing pyruvate–ferredoxin oxidoreductase (*pfo*), which is commonly found in anaerobic organisms. A part of acetyl-CoA is converted to acetate by generating ATP; the acetoacetyl-CoA (C4) is produced by condensing 2 mol of the remaining acetyl-CoA (C2), and further is catalyzed toward crotonyl-CoA. In Route B, *M. elsdenii* synthesizes oxaloacetate (C4) from pyruvate (C3) and HCO_3_ (C1) via pyruvate carboxylate (*pyc*). Succinate is then produced from oxaloacetate by running the TCA cycle in a reductive (counterclockwise) direction. Note that the reductive TCA cycle, from oxaloacetate to succinate, is also present in *C. acetobutylicum* [29]. Crotonyl-CoA (C4) is produced reductively from succinate via succinate semialdehyde. Butyryl-CoA is then synthesized from crotonyl-CoA through the butyryl-CoA dehydrogenase and electron transfer flavoprotein complex (*bcd*/*etf*AB), and in turn elongated to hexanoyl-CoA (C6) by combining with acetyl-CoA.

Routes A and B are decomposing and composing pathways, respectively. In Route A, the C3 metabolite is divided into C1 and C2 metabolites. Two molecules of the C2 metabolite are then integrated to build a C4 metabolite. Thus, two molecules of the C1 metabolite are withdrawn to synthesize one molecule of the C4 metabolite. However, in Route B, the C3 metabolite is combined with the C1 metabolite to synthesize a C4 metabolite. Route A is less efficient with respect to the C-balance because of CO_2_ loss—one 6C product (hexanoic acid) requires three molecules of pyruvate (3C * 3 = 9C), while wasting three carbon atoms as CO_2_. However, a more comprehensive comparison of Routes A and B should be made in terms of other energetically expensive consumables such as ATP, acetyl-CoA, and reducing equivalents (NADH).

Pyruvate is divided into acetyl-CoA and CO_2_ via *pfo,* while reducing ferredoxin. A portion of CO_2_ is fixed with H_2_O in order to produce HCO_3_ and a proton via carbonate dehydratase, and the residual CO_2_ secretes it out of cell. While producing 1 crotonyl-CoA from 2 acetyl-CoA, 1 NADH is oxidized (reaction 1).

2 Acetyl-CoA + NADH + H^+^ → Crotonyl-CoA + NAD^+^ + CoA + H_2_O (1)

One crotonyl-CoA is synthesized from pyruvate through succinate in a reductive direction, while oxidizing 5 NADH and reducing 1 ferredoxin (Reaction 2).

Pyruvate + Acetyl-CoA + Fd _ox_ + 5 NADH + 2 H^+^ + ATP + HCO_3_ → Crotonyl-CoA + Acetate + Fd _red_ + 5 NAD^+^ + 3 H_2_O + ADP + Pi (2)

The oxidation of 2 NADH and the reduction of 1 ferredoxin is catalyzed by *bcd*/*etf*AB, while 1 crotonyl-CoA is converted to butyryl-CoA. The enzymes that catalyze for synthesizing hexanoyl-CoA from butyryl-CoA are the same as those that participated in butyryl-CoA synthesis from acetyl-CoA. Then, 2 reduced ferredoxin and 5 NAD^+^ are generated via the hexanoyl-CoA synthetic pathway from crotonyl-CoA according to Reaction 3 (Figure 2).

Crotonyl-CoA + Acetyl-CoA + 2 Fd _ox_ + 5 NADH → Hexanoyl-CoA + CoA + 2 Fd _red_ + 5 NAD^+^ + H_2_O + H^+^ (3)

Flux is driven through the NADH:ferredoxin oxidoreductase (*rnf*A-E) by reduced ferredoxin and NAD^+^ to regenerate the necessary NADH. The *rnf* complex couples proton translocation with the oxidation of ferredoxin and reduction of NAD^+^. The proton gradient, which is generated by the *rnf* complex, is a driving force for synthesizing ATP via ATPase (*atp*A-I). The resulting fluxes of Reactions 1, 2, and 3 allowed for the cell to make a balance of reduced ferredoxin and oxidized NAD in order to produce the required amounts of ATP and NADH. The excess electrons, which were not oxidized through the *rnf* complex, are delivered to H^+^ via hydrogenase (*hyd*) for H_2_ formation. The reduced molecular hydrogen (H_2_) is then secreted.

Acetate, which is produced from acetyl-CoA or during a reductive TCA cycle, acts as coenzyme A (CoA) acceptor, while butyric and hexanoic acid are converted from butyryl-CoA and hexanoyl-CoA, respectively. Therefore, it is also important to synthesize an adequate amount of acetate to produce the maximal yield of hexanoic acid in *M. elsdenii*.

### 3.6. Flux Ratio Analysis for Hexanoic Acid Production

**Figure 3:**
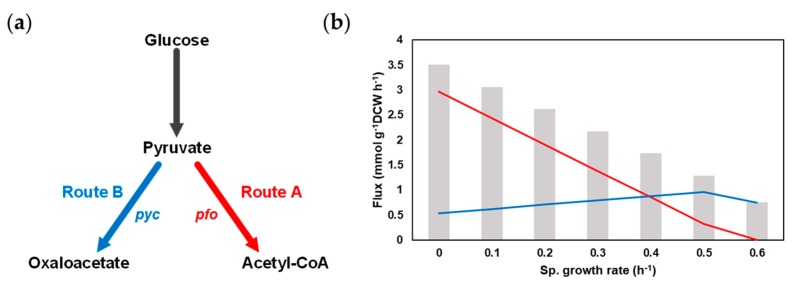
Flux ratio analysis for hexanoic acid synthetic pathways in *M. elsdenii*. (**a**) Manipulation of flux ratio with *pfo* and *pyc* reactions. (**b**) Flux distributions towards Routes A (Red) and B (Blue) at different specific cell growth rates.

As we have seen in the previous section, hexanoic acid synthetic metabolism is complicated in *M. elsdenii* since it is composed of many pathways such as 2 types of crotonyl-CoA synthesis, fatty acid elongation, CO_2_ fixation, electron transfer and ATP synthesis. In addition, the FBA result of *i*ME375 by fixing the glucose uptake rate of 5 mmol g^−1^ DCW h^−1^ and maximizing the hexanoic acid synthetic reaction showed that 3.51 mmol g^−1^ DCW h^−1^ of hexanoic acid was produced by using both of the above-mentioned pathways. The flux values of Routes A and B were 2.97 mmol g^−1^ DCW h^−1^ and 0.54 mmol g^−1^ DCW h^−1^, respectively. In order to further investigate the effect of the specific cell growth rate and flux ratio of Route A to Route B on hexanoic acid productivity, we conducted the flux ratio analysis in different cell growing rates (see Materials and Methods Section 2.5; Figure 3a).

First, to check the production patterns of hexanoic acid with the cell growth rate changes, the hexanoic acid production was maximized while constraining the biomass formation reaction from 0.6, the maximum specific growth rate, to 0. The total hexanoic acid production rate was gradually enhanced while the specific cell growth rate declined (Figure 3b). Only Route B was utilized for hexanoic acid synthesis at 0.6 h^−1^. When the specific growth rate was shifted from 0.6 to 0.5 h^−1^, the hexanoic acid production was raised, as the flux via both Route A and B increased. On the other hand, the flux towards Route A was dramatically increased, while the flux towards Route B was slightly reduced when the specific growth rate diminished from 0.5 to 0 h^−1^.

Flux ratio analysis was further performed in each cell growth rate by changing the flux ratio of pyruvate carboxylase (*pyc*) and pyruvate:ferredoxin oxidoreductase (*pfo*) with nine different ratios (10:1, 5:1, 3:1, 2:1, 1:1, 1:2, 1:3, 1:5, and 1:10). The highest hexanoic acid production rate was maintained through most of the flux ratios (Supplementary Data S6), as phosphoenolpyruvate carboxykinase (*pck*) helps to form consistent flux through Route B. Unlike the results of other specific growth rates, at 0.6 h^−1^, a slight increase (6%) of hexanoic acid production was observed when setting the flux ratio as 5:1. Based on the result of the flux ratio analysis, we could guess that the hexanoic acid productivity may not be increased simply by the knockdown of *pfo* or the overexpression of *pyc*. Thus, in order to find the potential metabolic engineering targets for improving the productivity of the target metabolite, a comprehensive understanding of the microorganism and the *in silico* strain design methods would be needed.

Additionally, we simulated *M. elsdenii* utilizing lactate, malate, succinate, and butyric acid as additional carbon sources in order to investigate the effects of organic acids on hexanoic acid production and biomass formation in *M. elsdenii,* as it was isolated from rumen with abundant of organic acids. It was assumed that the same amount of additive organic acids are consumed as for glucose. For that, each organic acid was calculated to contain an equal number of carbon moles and was additionally constrained in the model. The simulation was conducted in both the growth and non-growth phases (Supplementary Data S7). In the growth phase, the biomass formation is highly affected by the malate additive, followed by succinate and lactate. Unexpectedly, adding butyric acid lowers the cell growth rate. At the same time, the hexanoic acid synthetic reaction is significantly influenced by butyric acid (about two-fold), followed by succinate, lactate, and malate. During the non-growth phase, hexanoic acid synthesis is enhanced by adding butyrate (almost two-fold), followed by succinate, malate, and lactate. As a result, succinate addition to a glucose medium can improve biomass formation and hexanoic acid production during both the growing and non-growing phase.

## 4. Conclusions

In this study, we reconstructed a genome-scale metabolic model of hexanoic acid synthetic bacteria *M. elsdenii*, *i*ME375, containing 375 genes, 521 reactions, and 443 metabolites. It was manually curated and validated with experimental data under various media conditions. The gene essentiality analysis revealed that acetate plays a crucial role as an electron acceptor. We also reported the existence of the bifurcated pathway to produce crotonyl-CoA from pyruvate in *M. elsdenii*. Furthermore, our *in silico* analysis results highlighted the yield of carbon and redox balance while synthesizing VFAs in a glucose minimal medium. Because of the above-mentioned factors, the highest hexanoic acid production was achievable when Routes A and B were balanced. While the cell is not growing, *M. elsdenii* mainly utilizes Route A (from pyruvate to crotonyl-CoA via acetyl-CoA), albeit the carbon yield of this flux is lower than in Route B (from pyruvate to crotonyl-CoA via oxaloacetate) for balancing the redox potentials. On the other hand, hexanoic acid is produced only through Route B at a maximum specific growth rate condition. The additional flux ratio analysis suggested that a simple gene knockdown or overexpression may not be suitable for improving the target metabolite in this system. In future, we expect to further improve our understanding of *M. elsdenii* when the metabolic model is integrated with high throughput -omics data.

## Figures and Tables

**Table 1:** Comparison of simulation data with experimental data in various conditions.

	*M. Elsdenii* CECT390		*M. Elsdenii* ATCC17753		*M. Elsdenii* ATCC25940	
	Exp ^1^	Sim	Exp ^2^	Sim	Exp ^3^	Sim
	(mmol g^−1^DCW h^−1^)					
Medium	Minimal with Lactate		Complex with Lactate		Complex with Glucose	
Cell growth (h^−1^)	0.06	0.06	0.19	0.21	0.03	0.03
Lactate	−28.28	−28.28	−51.45	−51.45	―	
Glucose	―		―		−2.62	−2.62
Acetate	8.76	8.76	15.56	36	―	
Propionic acid	17.61	16.7	3.37	3.71	―	
Butyric acid	0.03	0.03	8.31	9.14	0.66	0.35
Pentatnoic acid	1.01	0.9	―		―	
Hexanoic acid	―		―		1.8	1.5

^1^ Data from [24], ^2^ Data from [22], ^3^ Data from [23].

## Supplementary Materials

The following are available online at https://www.mdpi.com/2076-2607/8/4/539/s1. Supplementary data S1: Biomass composition of *Megasphaera elsdenii i*ME375 model. Supplementary data S2: Hypothetical ORF annotations based on BLASTp search. Supplementary data S3: Reactions and metabolites of *i*ME375. Supplementary data S4: SBML file of *i*ME375. Supplementary data S5: Homology test of VFA synthetic enzymes in *Megasphaera elsdenii* and *Clostridium acetobutylicum*. Supplementary data S6: Flux ratio analysis on hexanoic acid production with specific cell growth rate in *Megasphaera elsdenii*. Supplementary data S7: Effect of various carbon sources on hexanoic acid production.
