# Supplementary material for: Genome-Scale Metabolic Network Reconstruction and In Silico Analysis of Hexanoic acid Producing Megasphaera elsdenii
Source: Microorganisms. 2020 Apr 9;8(4):539. doi: 10.3390/microorganisms8040539 (PMC7232489; doi:10.3390/microorganisms8040539)
Supplement: Supplementary file 1 [file microorganisms-08-00539-s001.zip › Supplementary data 1.docx]

**Supplementary data 1: Biomass composition of *Megasphaera elsdenii* *i*ME375 model**

1. **Overall Composition**

Overall cellular composition data were obtained from *Escherichia coli* *i*AF1260 model [1].

**Cellular composition**

| **Components** | **Weight percentage** |
| --- | --- |
| Protein | 56.3 |
| RNA | 21 |
| DNA | 3.1 |
| Phospholipid | 9.3 |
| Peptidoglycan | 2.5 |
| Carbohydrate | 2.5 |
| Trace Components | 4.94 |
| Total | 99.64 |

1. **Protein Composition**

The amino acid compositions were adopted from previous study [2]. The composition of tryptophan and cysteine was taken from *Clostridium acetobutylicum* *Cac*MBEL489 [3]. Energy requirement for polymerization was taken from *E. coli* [1].

| **Amino acids** | **mmol/ gDCW** | **mmol/ g**  **protein** | **Source** |
| --- | --- | --- | --- |
| GLY | 0.380 | 0.675 | [2] |
| ALA | 0.488 | 0.867 | [2] |
| VAL | 0.245 | 0.435 | [2] |
| LEU | 0.277 | 0.492 | [2] |
| ILE | 0.171 | 0.304 | [2] |
| PRO | 0.188 | 0.334 | [2] |
| PHE | 0.141 | 0.250 | [2] |
| TYR | 0.114 | 0.202 | [2] |
| TRP | 0.022 | 0.040 | [3] |
| MET | 0.109 | 0.194 | [2] |
| CYS | 0.642 | 1.141 | [3] |
| SER | 0.201 | 0.357 | [2] |
| THR | 0.217 | 0.385 | [2] |
| HIS | 0.086 | 0.153 | [2] |
| LYS | 0.254 | 0.451 | [2] |
| ARG | 0.158 | 0.281 | [2] |
| ASN | 0.210 | 0.373 | [2] |
| ASP | 0.210 | 0.373 | [2] |
| GLN | 0.235 | 0.417 | [2] |
| GLU | 0.235 | 0.417 | [2] |
| Total | 4.204 |  | |

1. **DNA Composition**

The DNA composition of *M. elsdenii* was calculated from its genome sequence. The G+C content is 52.8%. This value is used to derive the ratio of nucleic acids in the DNA. The molecular weights in the following table is the weight if the nucleotide monophosphate minus the weight of water molecule, which is lost during esterification.

| **Nucleotide** | **DNA (mol/mol)** | **MW (g/mol)** | **DNA (g/mol)** | **DNA (g/g)** | **g/gDCW** | **mmol/gDCW** | **mmol/g**  **DNA** |
| --- | --- | --- | --- | --- | --- | --- | --- |
| dATP | 0.236 | 487.151 | 114.9676 | 0.2383 | 0.0062 | 0.0127 | 0.4103 |
| dCTP | 0.264 | 503.15 | 132.8316 | 0.2754 | 0.0072 | 0.0142 | 0.4590 |
| dTTP | 0.236 | 478.136 | 112.8401 | 0.2339 | 0.0061 | 0.0127 | 0.4103 |
| dGTP | 0.264 | 461.109 | 121.7328 | 0.2524 | 0.0066 | 0.0142 | 0.4590 |
| Sum | | | 482.3721 |  | | 0.0539 |  |

1. **RNA Composition**

The RNA composition of *M. elsdenii* was taken from *E. coli* which is consisted of 5% mRNA, 81% rRNA, 14% tRNA. Energy required for polymerization is adopted from *E. coli*.

| **Nucleotide** | **RNA (mol/mol)** | **MW (g/mol)** | **mmol/ g RNA** |
| --- | --- | --- | --- |
| dATP | 0.262 | 328.20 | 0.810 |
| dCTP | 0.200 | 304.18 | 0.618 |
| dUTP | 0.216 | 305.16 | 0.668 |
| dGTP | 0.322 | 344.20 | 0.995 |

1. **Phospholipid Composition**

The phospholipid of *M. elsdenii* is mainly composed of plasmalogen, but the synthetic pathway in anaerobic bacterium is undiscovered. Thus, we calculated fatty acids, fatty aldehydes and polar lipid as described in *C. acetobutylicum* *Cac*MBEL489 [3]. The composition of acyl, alk-1-enyl and plasmalogen was adopted from previous study [4].

Molar ratio of plasmalogens to total polar lipids 0.495

1. Acyl chain composition

| **Fatty acids** | **g/g**  **total acyl chain** | **MW (g/mol)** | **mmol/g**  **total acyl chain** | **mol/mol**  **total acyl chain** |
| --- | --- | --- | --- | --- |
| 12;1 | 0.047 | 197.2 | 0.238 | 0.065 |
| 14;0 | 0.019 | 227.4 | 0.084 | 0.023 |
| 15;0 | 0.011 | 241.4 | 0.046 | 0.012 |
| 16;0 | 0.189 | 255.4 | 0.740 | 0.202 |
| 16;1 | 0.074 | 253.4 | 0.292 | 0.080 |
| 17;0 | 0.024 | 269.4 | 0.089 | 0.024 |
| 17;cyc | 0.097 | 265.4 | 0.365 | 0.100 |
| 18;0 | 0.068 | 283.5 | 0.240 | 0.066 |
| 18;1 | 0.138 | 281.5 | 0.490 | 0.134 |
| 19;cyc | 0.315 | 293.5 | 1.073 | 0.293 |
| Average molecular weight | | 273.4 |  |  |

1. Alk-1-enyl chain composition

| **Fatty acids** | **g/g**  **total acyl chain** | **MW (g/mol)** | **mmol/g**  **total acyl chain** | **mol/mol**  **total acyl chain** |
| --- | --- | --- | --- | --- |
| 16;0 | 0.248 | 239.4 | 1.036 | 0.265 |
| 16;1 | 0.07 | 237.4 | 0.295 | 0.075 |
| 17;0 | 0.014 | 254.4 | 0.055 | 0.014 |
| 17;cyc | 0.209 | 249.4 | 0.838 | 0.214 |
| 18;0 | 0.077 | 267.5 | 0.288 | 0.074 |
| 18;1 | 0.2 | 265.5 | 0.753 | 0.192 |
| 19;cyc | 0.18 | 277.5 | 0.649 | 0.166 |
| Average molecular weight | | 255.5 |  | |

1. Average fatty acid and aldehyde composition (mol/total fatty acid or aldehyde) in phospholipids

| **Fatty acids and aldehyde** | **Ratio** | **Average MW** |
| --- | --- | --- |
| 12;1 | 0.033 | 197.2 |
| 14;0 | 0.012 | 227.4 |
| 15;0 | 0.006 | 241.4 |
| 16;0 | 0.233 | 247.5 |
| 16;1 | 0.078 | 245.5 |
| 17;0 | 0.019 | 262.0 |
| 17;cyc | 0.156 | 257.5 |
| 18;0 | 0.070 | 275.6 |
| 18;1 | 0.163 | 273.6 |
| 19;cyc | 0.230 | 285.6 |
| Sum | 1 |  |
| Average MW of total fatty acid and aldehyde | 262.2 |  |

1. Polar lipid composition

| **Polar groups** | **g/g**  **total polar group** | **MW** | | | **mmol/g** |
| --- | --- | --- | --- | --- | --- |
|  |  | **Backbone** | **The number of fatty acid chain** | **Total** |  |
| Phosphatidyl-ethanolamine | 0.562 | 181.128 | 2 | 705.6 | 0.797 |
| Phosphatidyl-serine | 0.348 | 226.124 | 2 | 750.6 | 0.464 |

1. **Peptidoglycan**

The backbone of peptidoglycan is composed of N-acetylmuramate and N-acetylglucosamine. Each N-acetylmuramate, which attached to L-alanine, Diaminopimelinic acid, D-alanine and D-glutamate, forms cross-link between amino acids. Molecular weight presented in the following table exclude the weight of water to account for bond formation.

| **Peptidoglycan** | **Molar ratio** | **MW (g/mol)** | **mmol/g**  **Peptidoglycan** |
| --- | --- | --- | --- |
| N-acetylmuramate | 1 | 275.26 | 1.106 |
| N-acetylglucosamine | 1 | 203.19 | 1.106 |
| L-alanine | 1 | 71.08 | 1.106 |
| Diaminopimelinic acid | 1 | 154.17 | 1.106 |
| D-alanine | 1 | 71.08 | 1.106 |
| D-glutamate | 1 | 129.12 | 1.106 |
| Total molecular weight |  | 903.89 |  |

1. **Carbohydrate**

The carbohydrate composition of *M. elsdenii* was complicated because it contains amylopectin-glycogen [5]. Therefore, we assumed that the molar ratio of amylopectin and glycogen is 1:1. Molecular weight presented in the following table exclude the weight of water to account for bond formation.

| **Carbohydrates** | **Molar ratio** | **MW (g/mol)** | **mmol/g**  **Carbohydrate** |
| --- | --- | --- | --- |
| Amylopectin | 1 | 162 | 3.09 |
| Glycogen | 1 | 162 | 3.09 |

1. **Trace component**

We assumed that the selected trace components are existed in equal ratio.

| **Trace components** | **Molar ratio** | **MW (g/mol)** | **mmol/g**  **Trace component** |
| --- | --- | --- | --- |
| NAD | 1 | 664.433 | 0.251 |
| NADP | 1 | 744.413 | 0.224 |
| COA | 1 | 767.535 | 0.217 |
| THF | 1 | 445.429 | 0.374 |
| FMN | 1 | 456.344 | 0.365 |
| FAD | 1 | 785.550 | 0.212 |

**REFERENCES**

1. Feist, A.M.; Henry, C.S.; Reed, J.L.; Krummenacker, M.; Joyce, A.R.; Karp, P.D.; Broadbelt, L.J.; Hatzimanikatis, V.; Palsson, B.Ø. A genome-scale metabolic reconstruction for *Escherichia coli* K-12 MG1655 that accounts for 1260 ORFs and thermodynamic information. *Mol. Syst. Biol.* **2007**, *3*, 121.

2. Wallace, R.J. Catabolism of amino acids by *Megasphaera elsdenii* LC1. *Appl. Environ. Microbiol.* 1986, *51*, 1141–1143.

3. Lee, J.; Yun, H.; Feist, A.M.; Palsson, B.; Lee, S.Y. Genome-scale reconstruction and *in silico* analysis of the *Clostridium acetobutylicum* ATCC 824 metabolic network. *Appl. Microbiol. Biotechnol.* **2008**, *80*, 849–862.

4. Johnston, N.C.; Goldfine, H. Effects of growth temperature on fatty acid and alk-1-enyl group composition of *Veillonella parvula* and *Megasphaera elsdenii* phospholipids. *J. Bacteriol.* **1982**, *149*, 567–575.

5. Cheng, K.J.; Brown, R.G.; Costerton, J.W. Characterization of a cytoplasmic reserve glucan from *Ruminococcus albus*. *Appl. Environ. Microbiol.* 1977.
