# Supplementary material for: Genome-Scale Metabolic Network Reconstruction and In Silico Analysis of Hexanoic acid Producing Megasphaera elsdenii
Source: Microorganisms. 2020 Apr 9;8(4):539. doi: 10.3390/microorganisms8040539 (PMC7232489; doi:10.3390/microorganisms8040539)
Supplement: Supplementary file 1 [file microorganisms-08-00539-s001.zip › Supplementary data 2.docx]

**Supplementary data 2: Hypothetical ORF annotations based on BLASTp search**

| **Locus** | **Original annotation** | **Suggesting annotation** | **Sources** |
| --- | --- | --- | --- |
| MELS_0101 | hypothetical protein MELS_0101 | lactate racemase | lactate racemase |
| MELS_0336 | Alcohol dehydrogenase | 4-hydroxybutyrate dehydrogenase | 4-hydroxybutyrate dehydrogenase |
| MELS_0409 | hypothetical protein MELS_0409 | ferredoxin | ferredoxin |
| MELS_0410 | hypothetical protein MELS_0410 | 4-hydroxybutyrate dehydrogenase | 4-hydroxybutyrate dehydrogenase |
| MELS_0745 | 2-hydroxyglutaryl-CoA dehydratase  D-component | lactoyl-CoA dehydratase subunit alpha | lactoyl-CoA dehydratase subunit alpha |
| MELS_0746 | R-phenyllactate dehydratase | lactoyl-CoA dehydratase subunit beta | lactoyl-CoA dehydratase subunit beta |
| MELS_0757 | carbon-nitrogen family hydrolase | beta-ureidopropionase | beta-ureidopropionase |
| MELS_1156 | aldehyde dehydrogenase | Succinate semialdehyde dehydrogenase | Succinate semialdehyde dehydrogenase |
| MELS_1860 | dihydroorotate dehydrogenase | dihydropyrimidine dehydrogenase | dihydropyrimidine dehydrogenase |
| MELS_2160 | aldehyde dehydrogenase | Succinate semialdehyde dehydrogenase | Succinate semialdehyde dehydrogenase |
| MELS_2182 | iron-containing alcohol dehydrogenase | 4-hydroxybutyrate dehydrogenase | 4-hydroxybutyrate dehydrogenase |
