# Supplementary material for: Genome-Scale Metabolic Network Reconstruction and In Silico Analysis of Hexanoic acid Producing Megasphaera elsdenii
Source: Microorganisms. 2020 Apr 9;8(4):539. doi: 10.3390/microorganisms8040539 (PMC7232489; doi:10.3390/microorganisms8040539)
Supplement: Supplementary file 1 [file microorganisms-08-00539-s001.zip › Supplementary data 5.docx]

**Supplementary data 5: Homology test of VFA synthetic enzymes in *Megasphaera elsdenii* and *Clostridium acetobutylicum***

**Enzyme homology test result is represented from 1 to 7 section. In section 8, clusters of genes which are involved in hexanoic acid synthetic pathway are shown. In section 9, it compares VFA synthetic pathway of *C. acetobutylicum* and *M. elsdenii*.**

1. **Acetyl-CoA conversion to crotonyl-CoA** (Butyryl-CoA conversion to trans-2-hexenoyl-CoA)

| **CDS** | **Predicted function** | **Gene name** | **AA identity with CA, %** |
| --- | --- | --- | --- |
| MELS_0260 | pyruvate-flavodoxin oxidoreductase | *pfo*1 | 60% to CA_C2229 |
|  |  |  | 54% to CA_C2499 |
| MELS_2044 | pyruvate-flavodoxin oxidoreductase | *pfo*2 | 63% to CA_C2229 |
|  |  |  | 58% to CA_C2499 |
|  |  |  |  |
| MELS_1025 | acetyl-CoA C-acetyltransferase | *thl*A1 | 68% to CA_C2873 |
|  |  |  | 65% to CA_P0078 |
| MELS_1026 | acetyl-CoA C-acetyltransferase | *thl*A2 | 58% to CA_C2873 |
|  |  |  | 57% to CA_P0078 |
|  |  |  |  |
| MELS_1448 | 3-hydroxybutyryl-CoA dehydrogenase | *hbd* | 62% to CA_C2708 |
|  |  |  | 26% to CA_C2009 |
|  |  |  |  |
| MELS_0462 | 3-hydroxybutyryl-CoA dehydratase | *crt*1 | 60% to CA_C2712 |
|  |  |  | 38% to CA_C2016 |
|  |  |  | 28% to CA_C2012 |
| MELS_1449 | 3-hydroxybutyryl-CoA dehydratase | *crt*2 | 59% to CA_C2712 |
|  |  |  | 38% to CA_C2016 |
|  |  |  | 28% to CA_C2012 |

1. **Succinate reduction to crotonyl-CoA**

| **CDS** | **Predicted function** | **Gene name** | **AA identity with CA, %** |
| --- | --- | --- | --- |
| MELS_2160 | succinate semialdehyde dehydrogenase | *suc*D | 31% to CA_C3657 |
| MELS_1156 | succinate semialdehyde dehydrogenase | *suc*D | 32% to CA_C3657 |
|  |  |  |  |
| MELS_0336 | 4-hydroxybutyrate dehydrogenase | 4*hb*D | 50% to CA_C1574 |
| MELS_0410 | 4-hydroxybutyrate dehydrogenase | 4*hb*D | 26% to CA_C1574 |
| MELS_2182 | 4-hydroxybutyrate dehydrogenase | 4*hb*D | 28% to CA_C1574 |
|  |  |  |  |
| MELS_0341 | acetyl-CoA hydrolase/transferase | *cat*1 | None |
|  |  |  |  |
| MELS_0342 | 4-hydroxybutyryl-CoA dehydratase | *abf*D | None |

1. **Crotonyl-CoA conversion to butyryl-CoA (trans-2-hexenoyl-CoA conversion to hexanoyl-CoA)**

| **CDS** | **Predicted function** | **Gene name** | **AA identity with CA, %** |
| --- | --- | --- | --- |
| MELS_0063 | acyl-CoA dehydrogenase | *bcd*1 | 43% to CA_C2711 |
|  |  |  |  |
| MELS_1181 | acyl-CoA dehydrogenase | *bcd*2 | 45% to CA_C2711 |
|  |  |  |  |
| MELS_2128 | acyl-CoA dehydrogenase | *bcd*3 | 57% to CA_C2711 |
|  |  |  |  |
| MELS_0461 | butyryl-CoA dehydrogenase | *bcd*4 | 56% to CA_C2711 |
|  |  |  |  |
| MELS_0747 | butyryl-CoA dehydrogenase | *bcd*5 | 55% to CA_C2711 |
|  |  |  |  |
|  |  |  |  |
| MELS_2126 | electron transfer flavoprotein subunit alpha | *etf*A1 | 52% to CA_C2709 |
|  |  |  | 43% to CA_C2543 |
| MELS_2127 | electron transfer flavoprotein subunit beta | *etf*B1 | 52% to CA_C2710 |
|  |  |  | 40% to CA_C2544 |
| MELS_1988 | electron transfer flavoprotein alpha/beta-subunit | *etf*A2 | 37% to CA_C2709 |
|  |  |  | 37% to CA_C2543 |
| MELS_1989 | electron transfer flavoprotein alpha/beta-subunit | *etf*B2 | 35% to CA_C2710 |
|  |  |  | 34% to CA_C2544 |

1. **Butyryl-CoA conversion to butyrate (Hexanoyl-CoA conversion to hexanoate)**

| **CDS** | **Predicted function** | **Gene name** | **AA identity with CA, %** |
| --- | --- | --- | --- |
| MELS_0415 | acetyl-CoA hydrolase/transferase | *cat*2 | None |
|  |  |  |  |
| MELS_0430 | acetyl-CoA hydrolase/transferase | *cat*3 | None |
|  |  |  |  |
| MELS_0437 | acetyl-CoA hydrolase/transferase | *cat*4 | None |

1. **Acetyl-CoA conversion to acetate**

| **CDS** | **Predicted function** | **Gene name** | **AA identity with CA, %** |
| --- | --- | --- | --- |
| MELS_0670 | phosphotransacetylase | *pta*1 | 47% to CA_C1742 |
|  |  |  |  |
| MELS_0889 | phosphotransacetylase | *pta*2 | 40% to CA_C1742 |
|  |  |  |  |
| MELS_2048 | acetate kinase | *ack*A | 55% to CA_C1743 |

1. **H_2_ formation**

| **CDS** | **Predicted function** | **Gene name** | **AA identity with CA, %** |
| --- | --- | --- | --- |
| MELS_0409 | ferredoxin |  | 44% to CA_C3621 |
| MELS_0894 | ferredoxin |  | 52% to CA_C0303 |
|  |  |  |  |
| MELS_0611 | (4Fe-4S)-binding protein |  | None |
| MELS_0755 | (4Fe-4S)-binding protein |  | 41% to CA_C3230 |
|  |  |  | 27% to CA_C0028 |
| MELS_1346 | (4Fe-4S)-binding protein |  | None |
|  |  |  |  |
| MELS_0444 | Fe-only hydrogenase maturation protein | *hyd*F | 55% to CA_C1651 |
|  |  |  | 33% to CA_C1711 |
|  |  |  | 28% to CA_C3734 |
|  |  |  | 28% to CA_C1295 |
|  |  |  |  |
| MELS_0888 | Fe-only hydrogenase | *hyd*A1 | 40% to CA_C0028 |
|  |  |  | 44% to CA_C1742 |
|  |  |  | 24% to CA_C3076 |
| MELS_1481 | Fe-only hydrogenase | *hyd*A2 | 41% to CA_C0028 |
|  |  |  | 26% to CA_C3230 |

1. **Membrane proteins involved in energy conservation**

| **CDS** | **Predicted function** | **Gene name** | **AA identity with CA, %** |
| --- | --- | --- | --- |
| MELS_0150 | electron transport complex protein | *rnf*B | None |
| MELS_0152 | electron transport complex protein | *rnf*A | None |
| MELS_0153 | electron transport complex protein | *rnf*E | None |
| MELS_0154 | electron transport complex protein | *rnf*G | None |
| MELS_0155 | electron transport complex protein | *rnf*D | None |
| MELS_0156 | electron transport complex protein | *rnf*C | None |
|  |  |  |  |
| MELS_1898 | F1Fo ATPase, subunit C | *atp*C | 37% to CA_C2864 |
| MELS_1899 | F1Fo ATPase, subunit D | *atp*D | 72% to CA_C2865 |
| MELS_1900 | F1Fo ATPase, subunit G | *atp*G | 35% to CA_C2866 |
| MELS_1901 | F1Fo ATPase, subunit A | *atp*A | 68% to CA_C2867 |
| MELS_1902 | F1Fo ATPase, subunit H | *atp*H | 25% to CA_C2868 |
| MELS_1903 | F1Fo ATPase, subunit F | *atp*F | 27% to CA_C2869 |
| MELS_1904 | F1Fo ATPase, subunit E | *atp*E | 33% to CA_C2870 |
| MELS_1905 | F1Fo ATPase, subunit B | *atp*B | 30% to CA_C2871 |

1. **CDS clusters for synthesizing hexanoic acid**

**
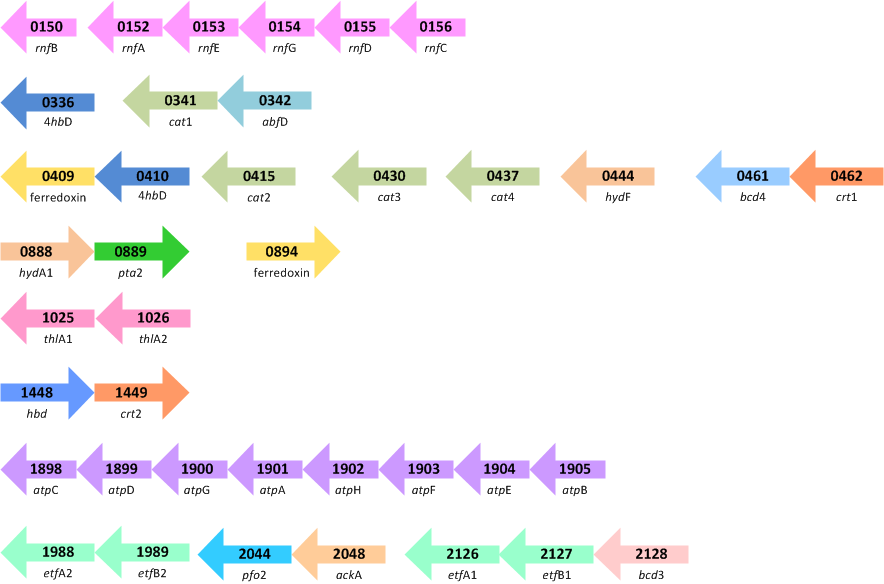
**

1. **Comparison of butyric and hexanoic acid synthetic pathway in *C. acetobutylicum* and *M. elsdenii***
