# Supplementary material for: Genome-Scale Metabolic Network Reconstruction and In Silico Analysis of Hexanoic acid Producing Megasphaera elsdenii
Source: Microorganisms. 2020 Apr 9;8(4):539. doi: 10.3390/microorganisms8040539 (PMC7232489; doi:10.3390/microorganisms8040539)
Supplement: Supplementary file 1 [file microorganisms-08-00539-s001.zip › Supplementary data 7.docx]

**Supplementary data 7: Effect of additional carbon source on hexanoic acid production**

- Flux distribution when malate is added during growth (left) and non-growth phase (right).

- Flux distribution when succinate is added during growth (left) and non-growth phase (right).

- Flux distribution when lactate is added during growth (left) and non-growth phase (right).

- Flux distribution when butyrate is added during growth (left) and non-growth phase (right).
